# Prospective screening of liver fibrosis in a primary care cohort using systematic calculation of fib-4 in routine results

**DOI:** 10.1371/journal.pone.0254939

**Published:** 2021-07-22

**Authors:** Philippe Halfon, Christelle Ansaldi, Guillaume Penaranda, Laurent Chiche, Patrick Dukan, Chloé Stavris, Anne Plauzolles, Frédérique Retornaz, Marc Bourliere

**Affiliations:** 1 Research & Development Department, Laboratoire Alphabio, Marseille, France; 2 Department of Internal Medicine, Hôpital Européen, Marseille, France; 3 Hepato-Gastroenterology Department, Hôpital Saint-Joseph, Marseille, France; Auburn University, UNITED STATES

## Abstract

**Background & aim:**

Liver fibrosis screening in primary care population is a major public health issue. The FIB-4 index is a simple non-invasive fibrosis test combining age, transaminases, platelets count, developed for the diagnosis of advanced fibrosis. The aim of our study was to evaluate the interest of liver fibrosis screening using systematic calculation of FIB-4 in routine blood analysis.

**Methods:**

Between December 2018 and May 2019, we conducted a prospective screening of liver fibrosis in 134 158 patients during a medical check-up including routine blood analysis. Among these patients, 29 707 had transaminases and platelets counts available and benefited from an automatic calculation of FIB-4. Results were obtained from 21 French clinical laboratories in the Bouches du Rhône region.

**Results:**

Among the 29 707 patients, 2161 (7.3%) had a high risk of advanced fibrosis (FIB-4>2.67). Individual investigation of patients with FIB-4>2.67 allowed to screen 1268 (1268/2161: 58.7%) patients who were not managed for any liver disease.

**Conclusions:**

This work demonstrates the interest of FIB-4 for the screening of liver fibrosis in primary care population. Although additional clinical validation study is required to determine the utility and applicability of Fib-4 to daily practice, our study strongly supports this easy-to-implement strategy using a simple Fib-4 measure resulting from the use of available routine test results.

## Introduction

Liver fibrosis screening in primary care population is a major public health issue. Among the causes of liver fibrosis, non-alcoholic fatty liver disease (NAFLD) is highly prevalent, affecting ∼25% of the population and is likely to increase further because of the obesity epidemic [1, 2]. NAFLD is typically asymptomatic, and therefore most of the patients remain undiagnosed. The only available data in French primary care population reported that 2.6% of patients were identified with advanced liver fibrosis (Constances Cohort) [3]. Various scoring systems/tools are available for the non-invasive assessment of liver fibrosis. Many require measurement of the aspartate aminotransferase (AST), while others include transient elastography (Fibroscan) and serum fibrosis tests including enhanced liver fibrosis (ELF) test, Fibrotest, Fibrometer, Hepascore, or APRI [4–6]. Neither NICE nor EASL-EASD-EASO guidelines make specific recommendations regarding when to use liver biopsy in NAFLD assessment, although the EASL-EASD-EASO, APASL, AASLD guidance advocate the approach of applying non-invasive methods first, to avoid biopsies in low-risk cases [7–10]. In addition, Newsome et al. recommend the use of FIB-4 in patients with potential NAFLD for the management of abnormal liver enzymes [11]. The UK NAFLD survey attempted to capture data on which tools are currently most widely used for non-invasive fibrosis assessment [8]. The survey indicates that primary care does not routinely perform any assessment of liver fibrosis, with only 7.9% routinely performing AST/ alanine aminotransferase (ALT) ratio in primary care.

Type 2 diabetes mellitus (T2D) was shown to be an independent risk factor for the development of non-alcoholic steatohepatitis (NASH) [12–14]. Screening this high-risk population using a simple noninvasive tool is mandatory in order to prevent and avoid hepatic and extrahepatic worse manifestations [15].

The aim of our study was to evaluate the interest of liver fibrosis screening using systematic calculation of FIB-4 in routine blood analysis (during which a hepatic blood test check-up and platelet count were prescribed).

## Patients and methods

Between December 2018 and May 2019, we conducted a prospective screening of liver fibrosis in 134 158 patients for whom routine blood tests were done during medical check-up addressed by primary care physicians. Fib-4 was systematically calculated in all patients for whom transaminases and platelets counts were available (ie. 29 707 patients).

The FIB-4 index is a simple non-invasive fibrosis test, combining age, transaminases, and platelet count, developed for the diagnosis of advanced fibrosis [16, 17]. The score contributes to the assessment of NASH, hepatic C virus (HCV) or cholestatic, and metabolic liver diseases. There are four variables considered: patient’s age, AST, ALT, and platelet count. The FIB-4 equation is $FIB4=\frac{AgexAST}{Platelet\;Count\;x\;\sqrt{ALT}}$. Cut-off values applied were >2.67 for high risk of advanced fibrosis, and low risk of advanced fibrosis was defined as <1.30 in patients aged under 65 years and <2.00 for patients aged 65 years or higher [18, 19].

Results were obtained from 21 French clinical laboratories located in Marseille, France. According to French regulations the study was approved by French ethics committees, (CPP Sud-Méditerranée II and Health Data Hub, approval number: I03140710192019https://www.health-data-hub.fr/projets/etude-fib4-depistage-de-la-fibrose-hepatique-laide-du-fib-4-calcule-automatiquement-lors). The ethics committee waived the need for a formal written informed consent from patient as this study was performed on clinical data retrieved from routine blood test, and thus no patient was specifically included for this study. As requested by French regulations, patients attending the clinical laboratory are informed that their biological results can be used for research purposes and that they are free to refuse (information annotated on the clinical laboratory report). All data were fully anonymized before analyses.

Age of patients, gender, FIB-4, platelet count, aspartate aminotransferase, alanine aminotransferase, and glycemia were retrieved from laboratory database. In case of multiple medical check-up of one individual patient, only the last one was considered for the analysis. All blood draws were performed under fasting conditions.

Two-sided Chi-square test was assessed to compare proportions at a significance level of 5%.

## Results

**Fig 1:**
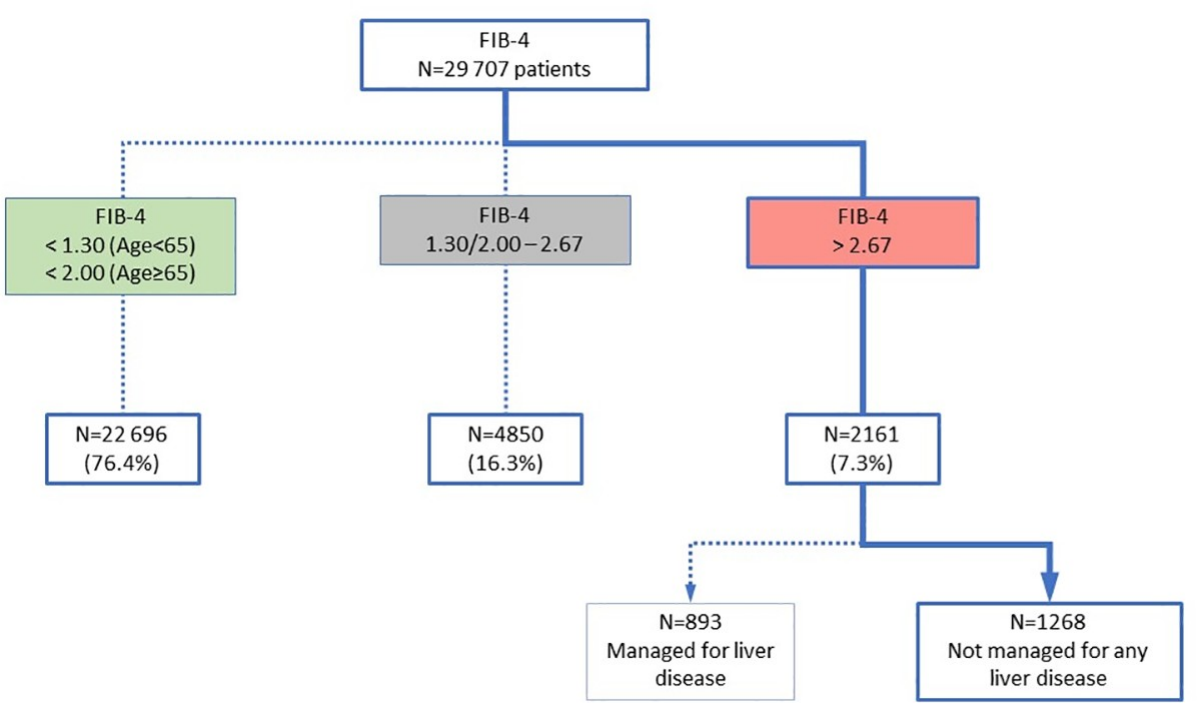
Study flowchart.

**Fig 2:**
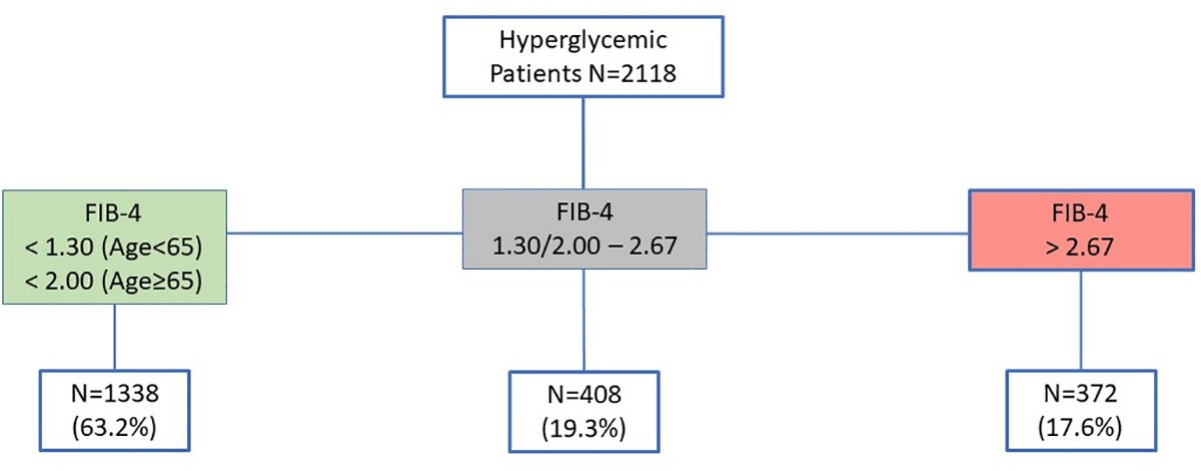
Flowchart of hyperglycemic patients.

Mean age of the 29 707 patients was 54 years old (Sd 21), 57.8% were females. Among these 29 707 patients and according to FIB-4 fibrosis staging, 2161 (7.3%) had a higher risk of advanced fibrosis (FIB-4>2.67), 22 696 (76.4%) had low risk of advanced fibrosis (FIB-4<1.3 in patients under 65 years old and FIB-4<2 in patients aged over 65 years old), and 4850 (16.3%) had an equivocal risk of fibrosis (age<65: FIB-4 ≥1.30 and FIB-4≤2.67, and age≥65: FIB-4≥2.00 and FIB-4≤2.67) (Fig 1). A subgroup of 1268 patients (1268/2161: 58.7%) was identified at high risk and potentially not diagnosed or followed up for a liver disease (Table 1): patients were arbitrarily considered as managed for liver disease if they were addressed to the laboratory by a hepato-gastroenterologist. Among the 2118 hyperglycemic patients (glycemia≥7 mmol∙L), 372 (17.6%) had a high risk of advanced fibrosis, 1338 (63.2%) had low risk of advanced fibrosis, and 408 (19.3%) had an equivocal risk of fibrosis (Fig 2). The rate of high risk of advanced fibrosis was higher in hyperglycemic patients compared with normoglycemic patients, 17,6% [CI95 16.0–19.3%] vs. 8.6% [7.9–9.2%] (p < .0001), respectively.

**Table 1:** Characteristics of the patients.

Characteristics	All Patients (n = 29 707)	Fib 4		Patients not managed for liver disease (n = 1268)
		≤2.67 (n = 27 546)	>2.67 (n = 2161)	
Gender–N(%)				
*Male*	12525 (42.2%)	11356 (41.2%)	1169 (54.1%)	685 (54.0%)
*Female*	17182 (57.8%)	16190 (58.8%)	992 (45.9%)	583 (46.0%)
Age–Mean (Sd) (years)	54 (21)	52 (20)	76 (14)	77 (13)
Platelet Count–Mean (Sd) (G/l)	255 (81)	262 (78)	167 (65)	161 (57)
Aspartate Aminotransferase–Mean (Sd) (IU/l)	29 (63)	25 (16)	86 (218)	60 (149)
Alanine Aminotransferase–Mean (Sd) (IU/l)	28 (56)	25 (31)	61 (172)	61 (172)

## Discussion

Our study provides an estimate of fibrosis prevalence in primary care practice and more importantly demonstrate the gap between patients with fibrosis who are undiagnosed and not managed for their liver disease and those who are diagnosed. This underscores the need for medical education on liver disease diagnosis and the utility of such tools to reinforce liver disease screening [20–25]. Two studies on primary care were performed in France: the first one (Constances) including 102 344 participants who were screened using the Fatty Liver index identified 16.7% of NAFLD patients; among them, 2.6% using the FORNS index were identified with liver fibrosis [3]; the second one reported by Poynard et al. in individuals >40 years old in two French Social Security centers reported a presumed prevalence of advanced fibrosis (2.8%) and cirrhosis (0.3%)(not confirmed by liver biopsy) [26].

In hyperglycemic patients, the present study identified 17.6% with a Fib-4 > 2.67 among 2118 patients with a Fib-4 > 2.67, which was very similar to the study of Kwok R et al. (1918 patients with type 2 diabetes from Hong Kong), and the prevalence of increased liver stiffness (>9.6 kPa, suggestive of stage ≥F3) was 18% [27]. Liver enzymes are normal in up to 80% of NAFLD patients, and therefore cannot identify patients with a liver fibrosis [28].

A limit of this study is that no confirmation was obtained from patients regarding the clinical follow-up of their potential liver disease. Another limitation of this study might be the cut-off used to define high risk of advanced fibrosis (ie. FIB-4>2.67). Princeps cut-off for advanced fibrosis (ie FIB-4>3.25) stills widely used, but is more stringent and was shown to be less sensitive [19]. The use of FIB-4 as a single non-invasive screening method might be criticizable. However, studies have shown a higher diagnostic accuracy of FIB-4 compared to other usual non-invasive methods for advanced fibrosis [17–19].

In conclusion, because even the most experienced clinicians may be unable to extract all the useful information from existing clinical and laboratory data, electronic clinical decision support represents an important tool to improve test result interpretation and its efficiency for converting diagnostic data into useful information [29]. Although additional clinical validation study is required to determine the utility and applicability of Fib-4 to daily practice, our study strongly supports this easy-to-implement strategy using a simple Fib-4 measure resulting from the use of available routine test results. That may represent an initial step to increase medical education and enhance fibrosis diagnosis.

## Supporting information

S1 Data (CSV)
